# Antarctic Penguin Biogeography Project: Database of abundance and distribution for the Adélie, chinstrap, gentoo, emperor, macaroni and king penguin south of 60 S

**DOI:** 10.3897/BDJ.11.e101476

**Published:** 2023-05-10

**Authors:** Christian Che-Castaldo, Grant Humphries, Heather Lynch

**Affiliations:** 1 Stony Brook University, ., United States of America Stony Brook University . United States of America

**Keywords:** Sampling event, ocean, Southern Ocean, Antarctica, birds, penguins, population abundance, biogeography

## Abstract

**Background:**

The Antarctic Penguin Biogeography Project is an effort to collate all known information about the distribution and abundance of Antarctic penguins through time and to make such data available to the scientific and management community. The core data product involves a series of structured tables with information on known breeding sites and surveys conducted at those sites from the earliest days of Antarctic exploration through to the present. This database, which is continuously updated as new information becomes available, provides a unified and comprehensive repository of information on Antarctic penguin biogeography that contributes to a growing suite of applications of value to the Antarctic community. One such application is the Mapping Application for Antarctic Penguins and Projected Dynamics (MAPPPD; www.penguinmap.com), a browser-based search and visualisation tool designed primarily for policy-makers and other non-specialists, and mapppdr, an R package developed to assist the Antarctic science community. This dataset contains records of *Pygoscelisadeliae*, *Pygoscelisantarctica*, *Pygoscelispapua*, *Eudypteschrysolophus*, *Aptenodytespatagonicus* and *Aptenodytesforsteri* annual nest, adult and/or chick counts conducted during field expeditions or collected using remote sensing imagery, that were subsequently gathered by the Antarctic Penguin Biogeography Project from published and unpublished sources, at all known Antarctic penguin breeding colonies south of 60 S from 01-11-1892 to 12-02-2022-02-12.

**New information:**

This dataset collates together all publicly available breeding colony abundance data (1979-2022) for Antarctic penguins in a single database with standardised notation and format. Colony locations have been adjusted as necessary using satellite imagery and each colony has been assigned a unique four-digit alphanumeric code to avoid confusion. These data include information previously published in a variety of print and online formats as well as additional survey data not previously published. Previously unpublished data derive primarily from recent surveys collected under the auspices of the Antarctic Site Inventory, Penguin Watch or by the Lynch Lab at Stony Brook University.

## Introduction

The Antarctic is one of the most rapidly changing regions of the planet, but the size and remoteness of the continent coupled with the logistical constraints of access have made it difficult to comprehensively map the distribution and abundance of species that breed or forage within its boundaries. While the Antarctic has been spared many of the anthropogenic disturbances faced by more developed parts of the planet, a confluence of factors leave the Antarctic no better off in terms of our global biodiversity targets (Chown et al. 2017); that Antarctica has been left out of the Convention on Biological Diversity’s Strategic Plan for Biodiversity makes it more likely that changes in Antarctic ecosystems will be lost to shifting baselines. Our knowledge of Antarctic biodiversity includes gaps across all scales of biological organisation, but conservation efforts have largely hinged on our understanding of species distributions and population dynamics. While management of Antarctic marine resources, such as krill, have focused their attention on trends in the circumpolar Adélie penguin, the emperor penguin and chinstrap penguin have also been highlighted as particularly vulnerable to climate change (Lee et al. 2022). Until recently, compilations of Antarctic penguin populations have been published irregularly as government-sponsored reports (e.g. Croxall and Kirkwood (1979), Woehler (1993)) or piecewise in expedition reports of particular geographic regions (e.g. Poncet and Poncet (1987)). Geographic locations lacked numerical precision and place names were highly idiosyncratic and often driven by geopolitical considerations (as is common in the Antarctic); this made it difficult to assemble coherent time series for a colony. Finally, different nationalities have focused on different geographic regions based on the locations of research stations and there has been relatively little incentive to share population data for these disparate regions.

Two major developments over the last decade have transformed our ability to track Antarctic penguin populations at the pan-Antarctic scale. First, the use of satellite imagery to identify penguin colonies has rapidly expanded over the last 15 years and satellite-based surveys have moved from a series of scattered demonstration projects to a well-established and well-documented means of both mapping the locations of colonies and tracking abundance at those colonies through time. This has had a tremendous impact on our ability to clarify the precise locations of colonies and, importantly, to document where penguins are not breeding. The combination of true presence and true absence (within detection thresholds) allows us an unprecedented overview of the biogeography of these species and satellites have allowed us to estimate abundance even in colonies too remote for direct survey. Satellites have been instrumental in completing the first global population estimates of emperor penguins (Fretwell et al. 2012), Adélie penguins (Lynch and LaRue 2014), chinstrap penguins (Strycker et al. 2020) and gentoo penguins (Herman et al. 2020).

Secondly, an effort initially sponsored by the US National Aeronautics and Space Administration (NASA) to develop a database and browser-based search tool called MAPPPD (Mapping Application for Penguin Populations and Projected Dynamics) precipitated an effort to comprehensively catalogue the precise location of all Antarctic penguin colonies and to assemble all publicly available census data published on the abundance at those colonies since 1979. It is worth noting that here we formally define and describe the organisation of this penguin biogeography dataset and that this effort is complementary to, but not identical with, the MAPPPD interface described by Humphries et al. (2017). MAPPPD is a user interface that allows easy access to the Antarctic penguin biogeography data, but there are now many means by which to discover, search, and download these data, each of which has been designed to reach different communities within the larger biodiversity sphere. The parallel development of the data (described here) and the various interfaces to it (e.g. MAPPPD, mappdr etc.), as well as the different schedules for updating these elements, required a modular approach to their development reflected in the separate publication of the database here described. MAPPPD data have already been used in a wide range of academic and applied management contexts, but MAPPPD data have not yet achieved full integration with global biodiversity assessments. This paper is intended to bridge that gap and presents a comprehensive and well documented dataset designed for easy integration with global assessments of biodiversity. We hope that this effort will allow Antarctica to become more integrated into international discussions regarding global biodiversity loss and potential solutions.

## General description

### Purpose

To provide open access penguin population census data to the general public.

## Project description

### Title

Antarctic Penguin Biogeography Project: Database of abundance and distribution for the Adélie, chinstrap, gentoo, emperor, macaroni and king penguin south of 60 S.

### Personnel

Christian Che-Castaldo, Heather Lynch, Grant Humphries.

### Funding

This project was initially funded by NASA Award NNX14AC32G under the NASA Ecosystem Forecasting programme. Continued funding for database expansion and updating provided by NASA Award 80NSSC21K1027 under the Biodiversity programme, a 2022 Pew Fellowship for Marine Conservation and the Institute for Advanced Computational Science at Stony Brook University.

## Sampling methods

### Study extent

**Figure 1.**
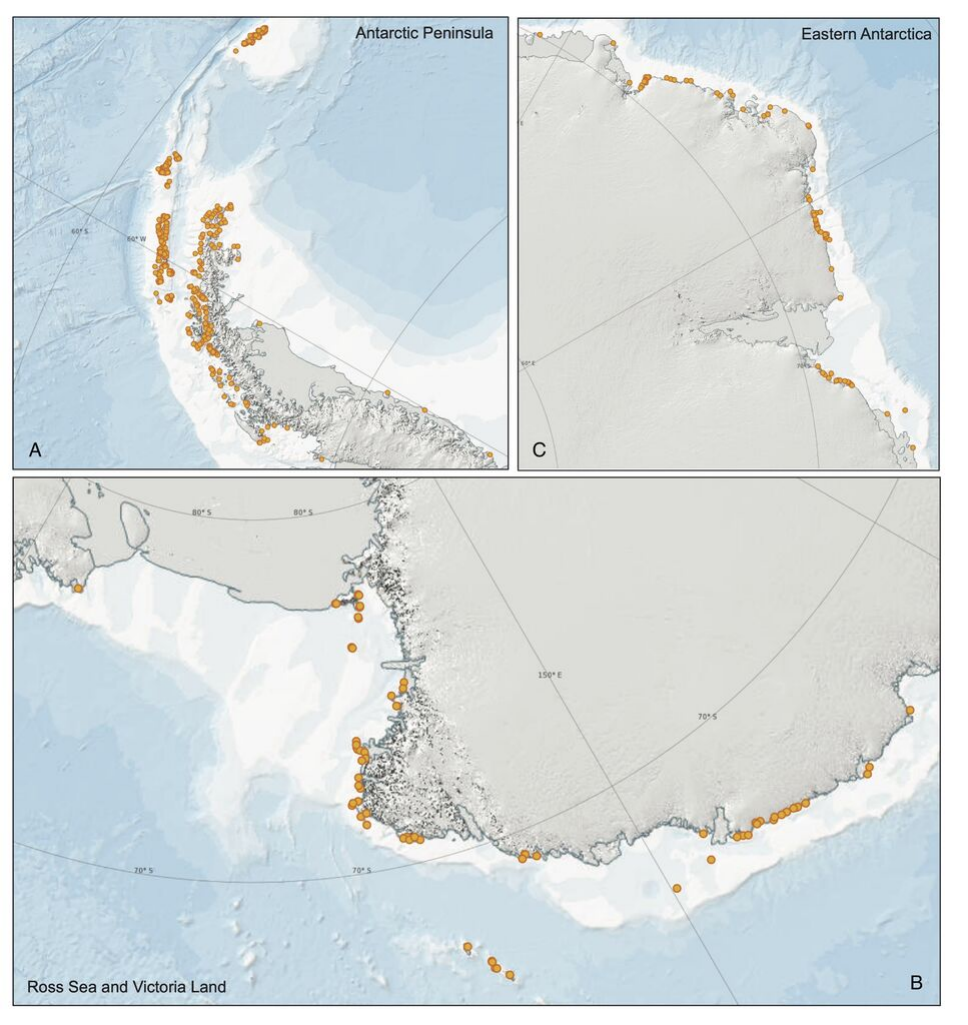
Areas in or along the A) Antarctic Peninsula, B) Ross Sea and Victoria Land and C) eastern Antarctica that contain the largest density of Antarctic penguin breeding colonies, whose locations are represented as orange circles. All breeding colony locations are taken from the Antarctic Penguin Biogeography Project: Database of abundance and distribution for the Adélie, chinstrap, gentoo, emperor, macaroni and king penguin south of 60 S event core archive.

This dataset describes the abundance and distribution of six species of Antarctic breeding penguins (Adélie, gentoo, chinstrap, emperor, macaroni and king) at all sites south of 60 S (Fig. 1). Data include all known historical data starting in 1979 and additional records as available prior to 1979.

### Sampling description

This database includes all known records of penguin breeding abundance and distribution south of 60 S. Data sources include peer-reviewed scientific manuscripts, expedition reports and other public datasets outside the scientific literature, management and policy documents and private communications. Abundance estimates are derived primarily from direct ground counting, imagery collected by remotely-piloted aircraft systems and satellite imagery. Additional data types include counts from aerial, ground or vessel-based photographs.

### Quality control

All records were validated. Coordinates were verified and plotted on a map to verify the actual geographical location corresponding to its locality. All scientific names were checked for typos and matched to the species information backbone of Worlds Register of Marine Species (http://marinespecies.org/) and LSID were assigned to each taxa as scientificNameID. Event date and time are validated to be in ISO 8601 format. To the extent necessary, authors were contacted to verify data that were ambiguous.

### Step description

Data contained within the core database include information collated from peer-reviewed scientific manuscripts, expedition reports and other public data outside the scientific literature, management and policy documents and private communications. These data contain information on breeding pairs of penguins only and does not contain information on non-breeding distributions or sightings-at-sea. The fundamental unit of this database is the breeding "site", which represents a population breeding on a single island or, in some cases, a discrete area of a larger landmass. In some cases, a geographically distributed population may be divided into multiple "sites" depending on the logistics of ground surveys, where each "site" can be accessed from a single landing location along the coastline. Rarely, several smaller nesting areas will be aggregated into a single "site" following historical precedent. Each site is associated with a unique four digit alphanumeric code, a name and a specific geographic location. Note that several species may inhabit a single "site". Data are ingested into the database manually and extensively checked against existing maps and records to ensure consistency. Due to the complexity of the geography and the different naming systems, site names may be changed between a published record and the database.

The database includes data on the number of nests (equivalently, breeding pairs), the number of chicks or the number of total adults. If multiple measures are available (a count of nests and also a count of chicks), the database will include all data points as separate entries. Each data point is associated with information on the survey date, the survey method, an estimate of the accuracy of each data point and a reference. In this database, we follow the five-point scale initially used by Croxall and Kirkwood (1979) and described in detail in Supplementary Materials 1 to Che-Castaldo et al. (2017). If the original data source followed the Croxall and Kirkwood scale or reported an uncertainty that could be translated into that scale, our database includes that precision value. Where no information on uncertainty is provided, we estimate the precision based on information provided in the original account.

Some data are not eligible to be included in this database. Data that are collected at a spatial scale other than the "site" cannot be included. All data must be "site-wide" census estimates. In keeping with our commitment to open-source data, data that are not in the public domain or remain in private collections unavailable to the public are also not included in this database. Data that cannot be verified and/or cannot be unambiguously assigned to a "site" are not included. Data on the presence of breeding pairs absent from a population estimate are included only when no survey data exist.

While traditional methods of surveying penguin colonies rely on the direct enumeration of penguin nests by ground-based researchers, more recent surveys have relied on various remote sensing methods. Remotely-piloted aircraft systems (RPAS; also known as drones, quadcopters or unmanned aerial vehicles) take photographs from a low-altitude flight over the colony and yield photographs sufficient for the individual enumeration of nests, chicks or adults. While the precision of such counts depends on the timing of the survey and the quality of the imagery, RPAS are capable of providing exceptionally precise counts and are often in the high accuracy category of N1. Satellite imagery can identify the areal extent of breeding colonies through the spectral properties of penguin guano and these estimates can be used to estimate the number of breeding pairs at a "site". As the precision of such estimates remains an active research question, all such estimates are assigned the lowest accuracy category of N5 (broadly defined as "order-of-magnitude").

In some cases, data from a single location in a given year are published in multiple venues. However, it is difficult to know whether these multiple outlets represent one actual sampling event because the same event may be associated with different occurrence or abundance records between sources, either because earlier mistakes were corrected or because data were re-analysed. It also can happen that a single site is surveyed multiple times by separate parties on the same day, arriving at different counts due to observation error. Our approach is to classify an event as a survey conducted at a specific site and time, using a specific sampling protocol, and reported in a specific publication (peer-reviewed article, policy document, thesis or other type of report), dataset or personal communication. From each event, there can be one or more occurrence or measurement records, depending on the number of species or life stages reported. In cases where we have information that data from a unique sampling event are published in multiple venues, we retain all records reported in publications and datasets and drop duplicate records if the duplicate event is based on personal communication. We allow this potential duplication because: 1) it is not clear which source is ultimately correct if counts differ between sources and 2) withholding records creates confusion with our end-users as to whether we have overlooked sources of data.

## Geographic coverage

### Description

Penguin breeding colonies located at or very near sea level distributed around the Antarctic continent and along the Antarctic Peninsula as well as on outlying islands in the Southern Ocean.

### Coordinates

-77.71 and -60.55 Latitude; -157.7 and 171.17 Longitude.

## Taxonomic coverage

### Taxa included

**Table:** 

Rank	Scientific Name	
kingdom	Animalia	
phylum	Chordata	
class	Aves	
order	Sphenisciformes	
family	Spheniscidae	
genus	*Pygoscelis*	
genus	*Eudyptes*	
genus	*Aptenodytes*	
species	*Pygoscelisadeliae*	
species	*Pygoscelisantarctica*	
species	*Pygoscelispapua*	
species	*Eudypteschrysolophus*	
species	*Aptenodytespatagonicus*	
species	*Aptenodytesforsteri*	

## Temporal coverage

### Notes

1892-11-01 through 2022-02-12

## Usage licence

### Usage licence

Open Data Commons Attribution License

### IP rights notes

This work is licensed under a Creative Commons Attribution (CC-BY) 4.0 License.

## Data resources

### Data package title

Antarctic Penguin Biogeography Project: Database of abundance and distribution for the Adélie, chinstrap, gentoo, emperor, macaroni and king penguin south of 60 S.

### Resource link

https://obis.org/dataset/b4be83a5-101d-4a82-80dd-b8a39c8026f2

### Alternative identifiers

https://ipt.biodiversity.aq/resource?r=mapppd_count_data, https://doi.org/10.48361/zftxkr, MAPPPD portal: https://www.penguinmap.com/mapppd/

### Number of data sets

1

### Data set 1.

#### Data set name

Antarctic Penguin Biogeography Project: Database of abundance and distribution for the Adélie, chinstrap, gentoo, emperor, macaroni and king penguin south of 60 S.

#### Data format

Darwin Core

#### Data format version

2.3

#### Description

The Antarctic Penguin Biogeography Project is an effort to collate all known information about the distribution and abundance of Antarctic penguins through time and to make such data available to the scientific and management community. The data are published as a standardised Darwin Core Archive and includes an event core and occurrence and eMoF extensions.

**Data set 1.** 

Column label	Column description
eventID	http://rs.tdwg.org/dwc/terms/eventID; An identifier for the set of information associated with a specific penguin sampling event, constructed as the concatenation of the citation key associated with the publication reporting the sampling event, the locationID, the eventDate and the vantage of the sampling event (vantage is explained more fully in the samplingProtocol field).
eventDate	http://rs.tdwg.org/dwc/terms/eventDate; The date-time or interval during which the penguin sampling event occurred. Here this refers to either the day, month(s) or year and conforms to ISO 8601-1:2019.
year	http://rs.tdwg.org/dwc/terms/year; The four-digit year in which the penguin sampling event occurred.
month	http://rs.tdwg.org/dwc/terms/month; The integer month in which the penguin sampling event occurred, if known.
day	http://rs.tdwg.org/dwc/terms/day; The integer day of the month in which the penguin sampling event occurred, if known.
decimalLatitude	http://rs.tdwg.org/dwc/terms/decimalLatitude; The geographic latitude (in decimal degrees, using the spatial reference system given in geodeticDatum) of the geographic centre of the penguin breeding colony that was sampled, specified in locationID.
decimalLongitude	http://rs.tdwg.org/dwc/terms/decimalLongitude; The geographic longitude (in decimal degrees, using the spatial reference system given in geodeticDatum) of the geographic centre of the penguin breeding colony that was sampled, specified in locationID.
coordinateUncertaintyInMetres	http://rs.tdwg.org/dwc/terms/coordinateUncertaintyInMeters; For an Adélie penguin breeding colony, this is computed as the average distance in metres between the polygon vertices from a polygon known to contain that colony and the colony site coordinates, as specified by decimalLatitude and decimalLongitude. Records for the remaining penguin species are set to NA as we lack colony polygons for their breeding colonies.
geodeticDatum	http://rs.tdwg.org/dwc/terms/geodeticDatum; The ellipsoid, geodetic datum or spatial reference system (SRS) upon which the geographic coordinates given in decimalLatitude and decimalLongitude are based, here EPSG:4326 (WGS84).
locality	http://rs.tdwg.org/dwc/terms/locality; The specific description of the penguin breeding colony, as standardised across all records.
locationID	http://rs.tdwg.org/dwc/terms/locationID; A unique identifier for the set of location information associated with a penguin breeding colony, as standardised across all records.
islandGroup	http://rs.tdwg.org/dwc/terms/islandGroup; The name of the island group (South Orkney Islands or South Shetland Islands) in which the penguin breeding colony is located, if applicable and NA otherwise.
waterBody	http://rs.tdwg.org/dwc/terms/waterBody; The name of the water body adjacent to the penguin breeding colony, here the Bellingshausen Sea, Ross Sea, Scotia Sea or Weddell Sea, if applicable and Southern Ocean otherwise.
higherGeography	http://rs.tdwg.org/dwc/terms/higherGeography; Geographic name from the Composite Gazetteer of Antarctic that represents a location less specific than the information captured in the locality term for the penguin breeding colony.
higherGeographyID	http://rs.tdwg.org/dwc/terms/higherGeographyID; Geographic ID associated with the name from the Composite Gazetteer of Antarctic that represents a location less specific than the information captured in the locality term for the penguin breeding colony.
country	http://rs.tdwg.org/dwc/terms/country; The name of the country or major administrative unit in which the penguin breeding colony occurs, here Antarctica.
countryCode	http://rs.tdwg.org/dwc/terms/countryCode; The standard code for the country in which the penguin breeding colony occurs, here AQ.
continent	http://rs.tdwg.org/dwc/terms/continent; The name of the continent which the penguin breeding colony occurs, here Antarctica.
samplingProtocol	http://rs.tdwg.org/dwc/terms/samplingProtocol; The names of, references to, or descriptions of the methods or protocols used during a penguin sampling event. Here we specify customised categories to distinguish penguins manually counted from the ground, air, or offshore vessel, UAV imagery, aerial or ground photograph or by other unknown means or penguin abundances statistically modelled from VHR, Landsat, or Sentinel satellite imagery.
occurrenceID	http://rs.tdwg.org/dwc/terms/occurrenceID; An identifier for the survey reporting the presence or absence (or count) of nests, adults or chicks for a given penguin species during a penguin sampling event. OcurrenceID was constructed as the concatenation of the citation key associated with the publication reporting the sampling event, the locationID, the eventDate, a four letter code unique to each penguin species and what was identified (adult, nest or chick). In cases where the same location was surveyed more than once for nests, chicks or adults of the same species on the same day and reported in the same publication, the occurrenceIDs are appended with integer counters to distinguish them from one another.
basisOfRecord	http://rs.tdwg.org/dwc/terms/RelatedBasisOfRecord; The nature of the survey, with human observation for surveys conducted from ground, aircraft or offshore vessel and machine observation for surveys or statistical analyses conducted of photographs or from UAV or satellite imagery, even though these latter surveys or analyses are also performed by humans.
type	http://rs.tdwg.org/dwc/terms/DwCType; The nature of the survey, where data from published peer-reviewed scientific studies, books, book chapters, conference proceedings or government reports are categorised as text, data from personal communications between two parties are categorised as events and data, either unpublished or published using a DOI, but not associated with a scientific study or other publication, are categorised as datasets.
kingdom	http://rs.tdwg.org/dwc/terms/kingdom; Animalia
phylum	http://rs.tdwg.org/dwc/terms/phylum; Chordata
class	http://rs.tdwg.org/dwc/terms/class; Aves
order	http://rs.tdwg.org/dwc/terms/order; Sphenisciformes
family	http://rs.tdwg.org/dwc/terms/family; Spheniscidae
genus	http://rs.tdwg.org/dwc/terms/genus; The penguin genus recorded during a penguin sampling event, here *Aptenodytes*, *Eudyptes* or *Pygoscelis*.
taxonRank	http://rs.tdwg.org/dwc/terms/taxonRank; species
scientificName	http://rs.tdwg.org/dwc/terms/scientificName; The scientific name of the penguin species recorded during a penguin sampling event, here *Pygoscelisadeliae*, *Pygoscelisantarctica*, *Pygoscelispapua*, *Eudypteschrysolophus*, *Aptenodytespatagonicus* or *Aptenodytesforsteri*.
scientificNameID	http://rs.tdwg.org/dwc/terms/scientificNameID; Identifier for the nomenclatural details of a scientific name taken from here: https://www.marinespecies.org.
scientificNameAuthorship	http://rs.tdwg.org/dwc/terms/scientificNameAuthorship; The authorship information for the scientificName formatted according to the conventions of the applicable nomenclaturalCode.
vernacularName	http://rs.tdwg.org/dwc/terms/vernacularName; Common or vernacular names of penguin species.
occurrenceStatus	http://rs.tdwg.org/dwc/terms/occurrenceStatus; A statement about the presence or absence of a given penguin species and life stage during a penguin sampling event.
lifeStage	http://rs.tdwg.org/dwc/iri/lifeStage; The life stage of the penguin species recorded during a penguin sampling event. Here, this is either adult or chick, where chicks are age 0 juveniles, but older than newborns.
organismRemarks	http://rs.tdwg.org/dwc/terms/organismRemarks; A comment about chicks defining them as age 0 juveniles, but older than newborns.
reproductiveCondition	http://rs.tdwg.org/dwc/iri/reproductiveCondition; The reproductive condition of the individuals in the occurrence record and is either reproductive for adults or non-reproductive for chicks.
organismQuantity	http://rs.tdwg.org/dwc/terms/organismQuantity; The total number of indviduals (adult or chicks) or nests (adults) at the penguin breeding colony during the survey determined from manual counts or statistically estimated from satellite imagery. This may be NA if only a presence / absence survey was conducted.
organismQuantityType	http://rs.tdwg.org/dwc/terms/organismQuantityType; The type of quantification system used for the quantity of organisms and is either nests (for adults) or individuals (for adults or chicks).
datasetName	http://rs.tdwg.org/dwc/terms/datasetName; The name identifying the dataset from which the record was derived, if the type was dataset.
language	http://purl.org/dc/terms/language; Language of the resource, here the text, event or dataset specified in the type field.
associatedReferences	http://rs.tdwg.org/dwc/terms/associatedReferences; A unique identifier of the literature associated with the Occurrence, here in the form of a citation.
measurementID	http://rs.tdwg.org/dwc/terms/measurementID; An identifier for the survey reporting counts of nests, adults or chicks for a given penguin species during a penguin sampling event, here identical to occurrenceID.
measurementTypeID	The nature of the measurement, here relying on codes from the NERC vocabulary server (NVS). We mapped an OrganismQuantityType of nests to a measurementType of breeding pairs and an OrganismQuantityType of individuals to a measurementType of individuals.
measurementType	http://rs.tdwg.org/dwc/terms/measurementType; The nature of the measurement, here either the the total number of nests (equivalently, breeding pairs) or the total number of chicks or adults.
measurementValue	http://rs.tdwg.org/dwc/terms/measurementValue; The total number of indviduals (adult or chicks) or nests (adults) at the penguin breeding colony during the survey determined from manual counts or statistically estimated from satellite imagery, here identical to OrganismQuantity.
measurementUnitID	The units of the measurement, here relying on codes from the NERC vocabulary server (NVS), here being dimensionless.
measurementUnit	http://rs.tdwg.org/dwc/terms/measurementUnit; The units associated with the measurementValue, here left blank.
measurementAccuracy	http://rs.tdwg.org/dwc/terms/measurementAccuracy; The description of the potential error associated with the measurementValue, here using a five point scale that defines the measurement error associated with a count. This accuracy category is decided by the observer and not estimated from data. See the Sampling Methods Step Description above for more details.

## Additional information

Che-Castaldo C, Humphries G, Lynch H (2023): Antarctic Penguin Biogeography Project: Database of abundance and distribution for the Adélie, chinstrap, gentoo, emperor, macaroni and king penguin south of 60 S. v.2.2. SCAR - AntOBIS. Dataset/Samplingevent. https://doi.org/10.48361/zftxkr, https://doi.org/10.48361/zftxkr
